# Associations between multimorbidity and neuropathology in dementia: a case for considering functional cognitive disorders, psychiatric illness, and dementia mimics

**DOI:** 10.1192/bjp.2024.25

**Published:** 2024-04-08

**Authors:** Calum A Hamilton, Fiona E Matthews, Johannes Attems, Paul C Donaghy, Daniel Erskine, John-Paul Taylor, Alan J Thomas

**Affiliations:** Newcastle University, United Kingdom

**Keywords:** Multimorbidity, Neuropathology, Dementia, BDR

## Abstract

**Background:**

Multimorbidity, the presence of two or more health conditions, has been identified as a possible risk factor for clinical dementia. It is unclear whether this is due to worsening brain health and underlying neuropathology, or other factors. In some cases, conditions may reflect the same disease process as dementia (e.g. Parkinson’s disease, vascular disease), in others, conditions may reflect a prodromal stage of dementia (e.g. depression, anxiety and psychosis).

**Aims:**

We aimed to assess whether multimorbidity in later life was associated with more severe dementia-related neuropathology at autopsy.

**Method:**

We examined antemortem and autopsy data from 767 brain tissue donors from the United Kingdom, identifying physical multimorbidity in later life, and specific brain-related conditions. We assessed associations between these purported risk factors and dementia-related neuropathological changes at autopsy (Alzheimer’s disease related neuropathology, Lewy pathology, cerebrovascular disease, and limbic-predominant age-related TDP43 encephalopathy) with logistic models.

**Results:**

Physical multimorbidity was not associated with greater dementia-related neuropathological changes. In the presence of physical multimorbidity, clinical dementia was less likely to be associated with Alzheimer’s disease pathology. Conversely, conditions which may be clinical or prodromal manifestations of dementia-related neuropathology (Parkinson’s disease, cerebrovascular disease, depression, and other psychiatric conditions) were associated with dementia and neuropathological changes.

**Conclusions:**

Physical multimorbidity alone is not associated with greater dementia-related neuropathological change; inappropriate inclusion of brain-related conditions in multimorbidity measures and misdiagnosis of neurodegenerative dementia may better explain increased rates of clinical dementia in multimorbidity.

**Data Set Information:**

Data were drawn from the Brains for Dementia Research study.

## Introduction

Multimorbidity, the co-occurrence of two or more long-term health conditions (LTCs), is common in older age and is a reported risk factor for dementia (1–3). However, the mechanisms of this are unclear. Multimorbidity may contribute to dementia risk through worsening underlying brain pathologies such as Alzheimer’s disease (AD), Lewy body disease (LBD), or cerebrovascular disease (CVD). An alternative explanation is that factors associated with multimorbidity may predispose people to cognitive impairments from other causes, such as functional cognitive disorders (FCDs).(4) Longitudinal cliniconeuropathological studies provide an opportunity to directly test these associations between multimorbidity and pathology seen at autopsy. We tested whether autopsy data from the UK Brains for Dementia Research (BDR) programme supported a hypothesised link between multimorbidity and dementia-related pathology.

## Method

### Participants

BDR participants were recruited from six sites across England and Wales (Newcastle, Manchester, Bristol, Cardiff, Oxford, and London), providing written informed consent for repeated research assessment, and for brain tissue donation. Research visits were facilitated by an informant (e.g. a family member or close friend), where available, and were conducted every 1-2 years after baseline until death. Prospective participants were identified through local research studies and clinical services, public research participation events, newsletters, and through online advertisement. This cohort was restricted for analysis to those who died aged at least 60 years and had provided at least one antemortem assessment to provide details of LTCs. Presence of dementia was ascertained through repeated administration of the Clinical Dementia Rating (CDR) at each visit, and defined as a CDR global score ≥1.

### Brain tissue donation

Brain tissue was donated post-mortem. Samples underwent standardised neuropathological assessment as previously described (5) to assess:

AD-related neuropathological change (6), rated by Thal phase of amyloid deposition,(7) Braak staging for neurofibrillary tangle (NFT) pathology,(8) and CERAD scoring of neuritic plaque density.(9)

LBD pathology staged by the Braak criteria.(10)

CVD according to the VCING criteria (11) (subcortical infarcts >10mm, moderate/severe occipital leptomeningeal cerebral amyloid angiopathy (CAA), or moderate/severe occipital white matter (WM) arteriolosclerosis).

Limbic-predominant age-related TDP-43 encephalopathy neuropathologic change (12) (LATE-NC).

Additional less-common pathologies were also assessed on a case-by-case basis, including argyrophilic grain disease,(13) corticobasal degeneration,(14) frontotemporal lobar degeneration,(15) and Creutzfeldt-Jakob disease. Since these had low prevalence in this cohort these were not included as modelled outcomes.

### Health data extraction

Data on LTCs were extracted from three complementary sources: ICD-10 codes were reported for each clinical diagnosis by BDR clinical research staff incorporating all information available (clinical research and primary care records, where available). Responses to specific health questions were identified from the Cambridge Mental Disorders of the Elderly Examination (CAMDEX) interview, again rated by BDR-trained clinical staff. Finally, free-text responses to the CAMDEX medical history questionnaire were systematically searched to identify LTCs not elsewhere reported. In the case of disagreement between clinically rated conditions and CAMDEX-reported conditions, the former (ICD-10 code) was treated as the most informative source. Those without CAMDEX data, or with any missing answers to the CAMDEX health questionnaire, were excluded. A single report of any given condition was sufficient to consider this as being present, so long as this corresponded to a formal long-term diagnosis (e.g. major depressive disorder would qualify as a long-term condition, but depressive symptoms reported in psychological testing alone would not).

### Defining multimorbidity

To enable stratification of groups by multimorbidity, key age-related LTCs from the Charlson Comorbidity Index (CCI) were identified, with ICD-10 codes corresponding to previous research.(16)

Modifications and supplements were made to the standard CCI to enable appropriate group comparisons. To prevent circular reasoning, diagnoses of clinical dementia (included in the standard CCI) were entirely excluded from multimorbidity classification.

In previous research, Parkinson’s disease, depression, and other mental disorders have also been included as indicators of multimorbidity.(1–3) We therefore also sought information on the presence of these conditions in addition to the CCI measures to test how the inclusion of these conditions affects the association between LTCs and dementia-related neuropathological change.

LTCs which could be clinical or prodromal manifestations of dementia-related neuropathological changes (Parkinson’s disease, or cerebral haemorrhage, infarct, stenosis, or other CVD, depression or other psychiatric condition) were not treated as indicators of multimorbidity in our primary analysis. These were instead grouped under a ‘brain comorbidity’ category and examined as separate predictors in secondary analyses.

Causes of death (e.g. fatal myocardial infarction) were not considered as indicators of multimorbidity, unless these had also been reported previously in life.

### Analysis

Associations between multimorbidity and neuropathological changes were assessed with Bayesian logistic models, adjusting for random differences between sampling sites, age at death, and both with and without APOE4 genotype for AD-related changes (available only for a subset of cases).

Staged neuropathological changes (Thal phase, Braak tangle stage, CERAD score, and overall VCING severity) were examined with ordinal models. Binary changes (Lewy body Braak stage ≥IV, LATE-NC, subcortical infarcts >10mm, CAA, and WM arteriolosclerosis) were estimated with Bernoulli models, as was clinical dementia as an outcome adjusting for age and education.

Models were estimated with the *brms* package for *R* software, as an interface to the *Stan* probabilistic programming language. Sensitivity analyses were undertaken with a range of flat, weakly informative and informative t-distributed priors, and with probit-link models to assess the robustness.

Sampling of posterior parameter estimates was undertaken with the No-U-Turn Sampler. Four chains were run in parallel for 2000 iterations (1000 warmup iterations) initially, with any non-convergence or inefficiency of chains diagnosed and addressed as required by increasing the target acceptance probability, or number of iterations, respectively. Models were then re-estimated with 6000 iterations to verify that convergence had been achieved. The effects of including APOE status was assessed in sensitivity analyses with missing data multiply imputed by Bayesian methods, which also assessed any effects of missingness in other variables.

### Ethics, inclusion and data availability

The authors assert that all procedures contributing to this work comply with the ethical standards of the relevant national and institutional committees on human experimentation and with the Helsinki Declaration of 1975, as revised in 2008. All procedures involving human subjects/patients were approved by the Health Research Authority North East – Newcastle & North Tyneside 1 Research Ethics Committee (18/NE/0124). Deidentified data from the Brains for Dementia Research programme are available to researchers through the United Kingdom Brain Bank Network, and Dementias platform UK.

## Results

### Primary analysis: physical multimorbidity

Seven hundred and sixty-seven participants had undergone autopsy and provided sufficient information to assess comorbid health conditions from the CCI; 328 were cognitively healthy or had mild cognitive impairment, 439 had clinical dementia based on antemortem assessment (i.e. without reference to pathological assessment). Overall, there was a mean interval of 4.0 years (SD=2.8) between the first observation and death, though this was shorter in those with dementia. The mean age at recruitment into the BDR cohort is 75.9 years (SD=8.5), however the available cohort with autopsy were older on average at initial assessment. (see Table 1). APOE status was known for 453 brain tissue donors, with 223 (49%) having one or more ε4 alleles.

The most common physical conditions reported were cancer (n=261), and diabetes (n=98), with all other assessed conditions being relatively more uncommon (see Table 2).

**Figure 1:**
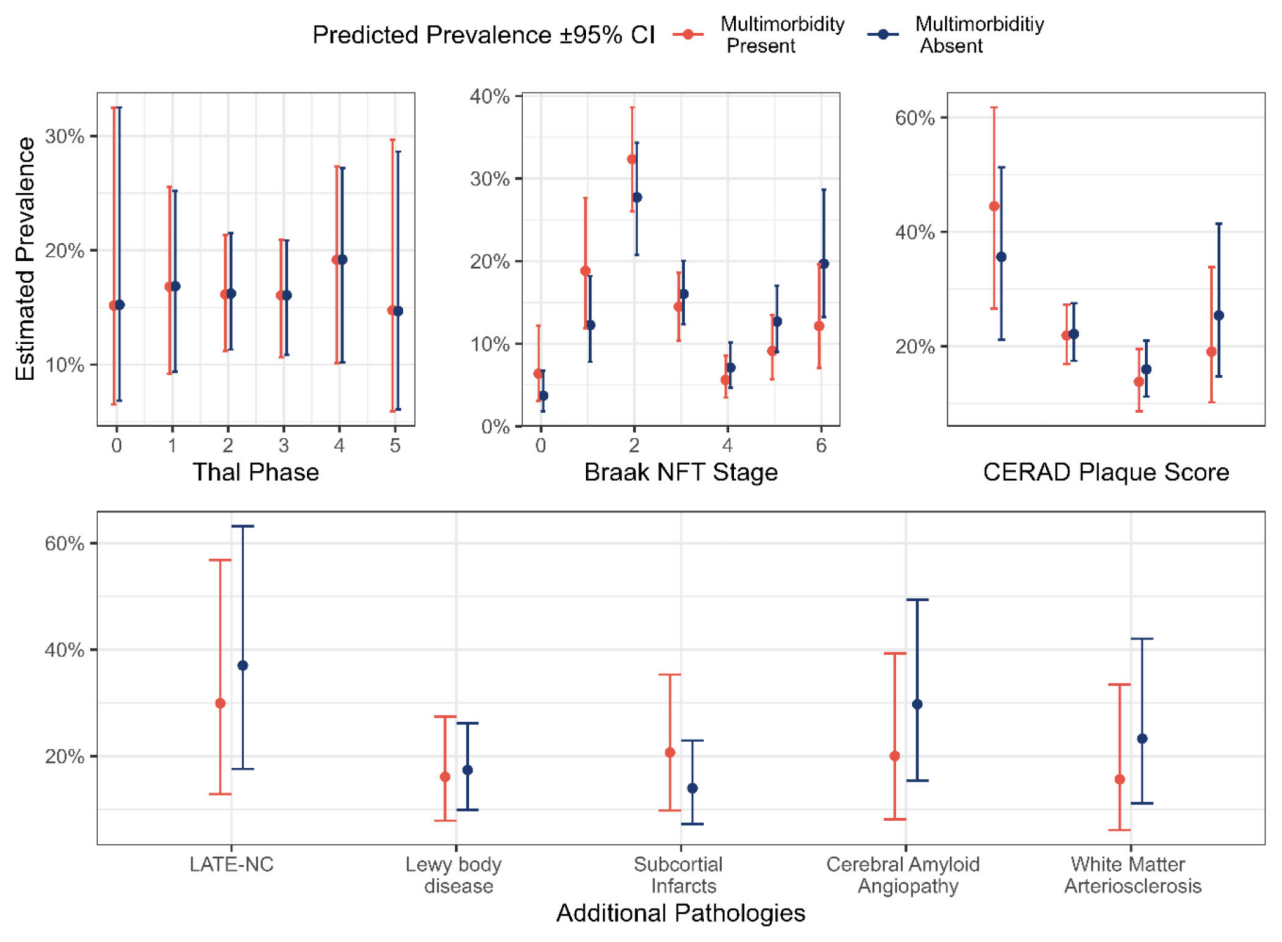
Associations between physical multimorbidity and key neuropathological changes.

When examining individual neuropathological criteria, there was little evidence of any association between physical multimorbidity and neuropathological changes (see Figure 1).

There was no clear association overall between physical multimorbidity and Aβ pathology rated by Thal phase (Odds Ratio (OR), 95%CI=1.01, 0.66–1.56), or CERAD score (OR=0.59, 0.44–1.11).

There was an overall negative association between multimorbidity and severity of Braak tangle pathology (OR=0.56, 0.37–0.84): those with multimorbidity had higher rates of lower Braak tangle staging (stages 1 or 2 in particular, in which the likelihood of AD pathology contributing to clinical symptoms is low regardless of Aβ pathology level), and lower rates of the highest Braak tangle stage.

There was also no clear evidence of a positive association between multimorbidity and LB pathology (OR=0.91, 0.48–1.65), LATE-NC (OR=0.73, 0.39-1.32) or CVD (Infarcts OR=1.61, 0.76–3.22; CAA OR=0.59, 0.32–1.06; WM arteriolosclerosis OR=0.62, 0.29–1.24; Overall VCING OR=0.98, 0.59-1.60).

The majority of participants reported at least one LTC in addition to dementia (where present). There was a higher rate of cancer in those who were dementia free, and none of the primary long-term conditions were clearly more common in those with dementia than those without (see Table 2).

When examining multimorbidity as a possible moderator of the relationship between overall AD-related pathology and presence of dementia, presence of multimorbidity weakened the relationship between the diagnosis of clinical dementia and the presence of Alzheimer’s pathology. That is, in those with multimorbidity, clinical dementia was less likely to be associated with Alzheimer’s pathology, compared to those without multimorbidity (see Supplementary Figure S1).

### Secondary analysis: brain comorbidities

We conducted several secondary exploratory analyses to test the effects of including different indicators of multimorbidity which have been included in previous research. These brain comorbidity measures included conditions which may be clinical or prodromal manifestations of dementia-related neuropathological changes: Parkinson’s disease and clinical diagnosis of cerebrovascular disease (which can directly cause clinical dementia), and psychiatric disorders which can have direct cognitive effects or can be prodromal to dementia (depression and non-depressive mental health conditions (anxiety, psychosis)). Personality and stress disorders were also examined as in previous studies; there were no cases of personality disorder reported, and a single case with post-traumatic stress disorder reported.

In contrast to the physical multimorbidity measures, multimorbidity of brain LTCs was clearly associated with substantially increased risks of dementia (see Supplementary Figure S2). This effect seemed to be driven primarily by Parkinson’s disease, depression and mental disorders: presence of any of these was associated with greater odds of dementia (OR=1.8, 1.2-3.0), which increased further as more predictors were observed (see Supplementary Figure S1A).

Examining the association with neuropathological changes, the individual and combined presence of Parkinson’s disease, depression, and non-depressive mental disorders were associated with increasing risks of Lewy body pathology specifically as more of these conditions were observed (see Supplementary Figure S1B), and adjusting for presence of Lewy body pathology largely attenuated the association between these conditions and clinical dementia (OR=1.49, 0.85–2.58). These conditions were not evidently associated with AD-related neuropathological changes, nor any measures of cerebrovascular pathology.

### Psychiatric multimorbidity

Finally, we assessed whether excluding Parkinson’s disease as an indicator of multimorbidity, while retaining depressive and non-depressive mental disorders, was sufficient to remove the association between dementia and Lewy body pathology. The association between mental health conditions and clinical dementia remained (OR=1.74, 1.03–2.99 for presence of one; 3.03, 1.06–8.94 for multimorbid depressive and non-depressive mental disorders; see Supplementary Figure S1C). The association with Lewy body pathology however was not supported in the absence of Parkinson’s disease (OR=1.58, 0.86–2.76 for presence of one; OR=2.48, 0.73–7.61 for psychiatric multimorbidity; see Supplementary Figure S1D), and there remained no clear relationship between these and Alzheimer’s disease pathology, nor cerebrovascular pathology.

While there was a reasonably strong separate association between dementia-related LTCs and dementia, directly incorporating these as indicators of multimorbidity was not sufficient to cause a positive association between overall multimorbidity and clinical dementia in this cohort as brain comorbidities were less common than physical comorbidities.

All analyses showed good convergence of sampling chains with all $\hat{R}$ values <1.01 and sufficient effective sample sizes. Sensitivity analyses tested the influence of prior choices on the outcome testing flat, weakly informative, and informative priors (anticipating a positive association between multimorbidity and neuropathological change, consistent with previously-reported associations with dementia). These analyses did not meaningfully change the results for any of the considered clinical or neuropathological outcomes, suggesting that the findings were not simply dictated by the influence of the prior, nor do they reflect a lack of data (in which case the informative prior would have the greatest influence); the data were robustly incompatible with a positive association between primary multimorbidity measures and dementia-related neuropathological change. We also assessed the impact of missing pathological data (see Supplementary Table S1) or APOE status with imputed datasets, which similarly did not change any findings.

Additional sensitivity analyses sought to examine the robustness of the choice of link function: probit models provided similar results to those presented here, with slightly attenuated risk ratios but narrower confidence intervals.

## Discussion

We tested whether multimorbidity would be associated with greater dementia-related neuropathology in this moderately-sized UK cohort. We found no evidence of a positive association between physical multimorbidity and dementia-related neuropathological changes. Physical multimorbidity weakened, rather than strengthened, the association between clinical dementia diagnosis and AD-related pathology.

In contrast, the occurrence or co-occurrence of specific LTCs which may be clinical or prodromal manifestations of dementia-related pathology – Parkinson’s disease, cerebrovascular disease, depression, and other psychiatric disorders – was positively associated with rates of clinical dementia and corresponding Lewy body pathology.

**Figure 2:**
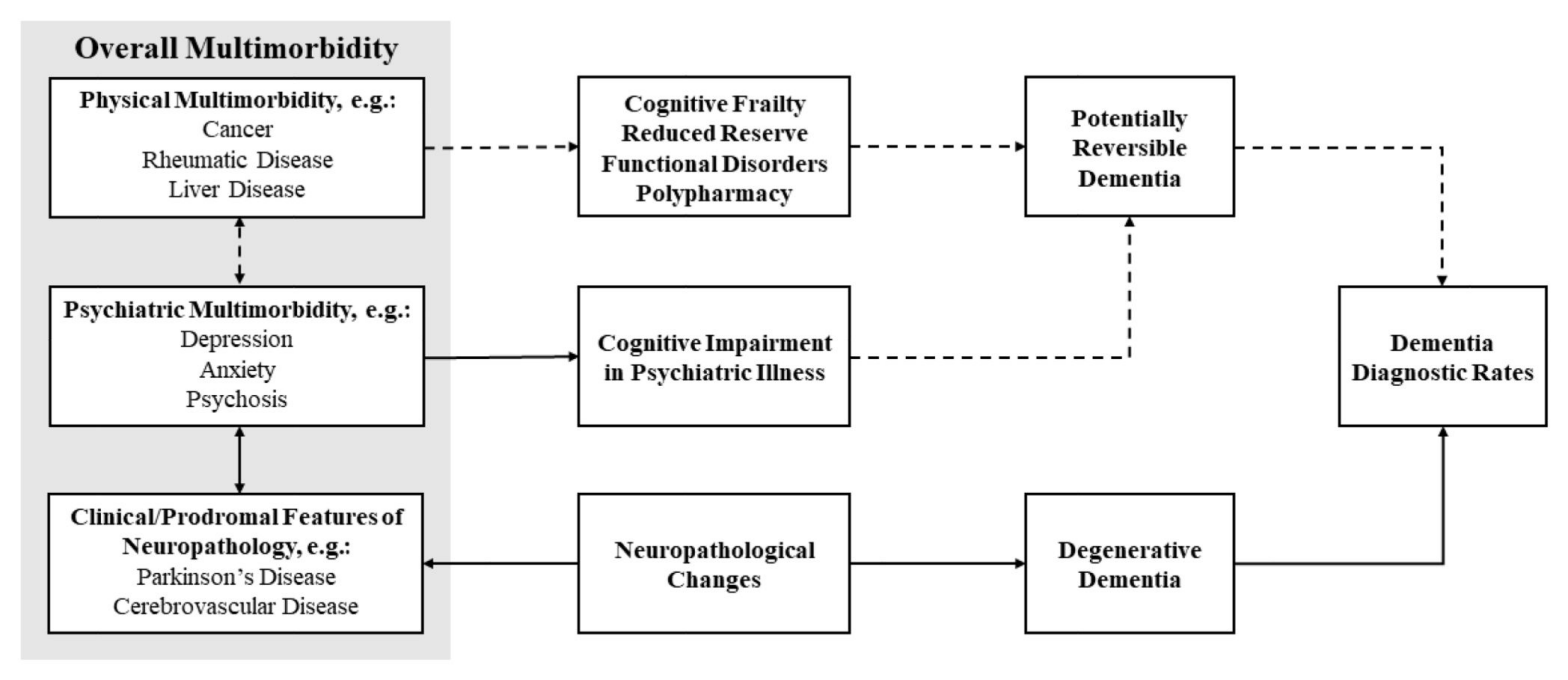
Theorised pathways by which subcategories of multimorbidity might result in greater rates of dementia diagnosis. Solid lines indicate pathways supported by presented data, dashed lines indicate theorised explanations which could remain consistent with previous research findings.

This does not support the hypothesised link between overall multimorbidity and dementia-related pathology, such as AD, and suggests mechanisms other than increasing dementia-related pathology may account for the reported relationship between overall multimorbidity and clinical dementia (see Figure 2).

Key considerations for interpreting our findings in research context include the selection of appropriate indicators of multimorbidity for dementia risk prediction, the differentiation of sustained, progressive dementias from transient cognitive complaints, the presence of cognitive symptoms as a direct consequence of illness, and the possible role of primary psychiatric conditions.

### Selection of multimorbidity indicators in dementia

Multimorbidity is not operationalised in a consistent manner across studies. Brain comorbidities (Parkinson’s disease, stroke/cerebrovascular disease and primary psychiatric disorders) have previously been treated as risk factors for dementia alongside physical LTCs.(1) This may be problematic, as brain comorbidities such as these have a different causal relationship with both dementia, and its associated pathologies, being brain conditions and in some cases (Parkinson’s disease, cerebrovascular disease) caused by dementia-related neuropathologies.

Consistent with this, we directly assessed brain multimorbidity separately and found that, unlike physical (non-brain) multimorbidity, this had a positive relationship with clinical dementia and associated pathology particularly due to the inclusion of Parkinson’s disease. In dementia risk factor studies, inclusion of Parkinson’s disease alongside other multimorbidity measures is likely to confound findings, given that the Lewy body disease underlying this is also a dementia pathology.

### Diagnosis of dementia across settings

The diagnosis of clinical dementia does not necessarily reflect the presence of neurodegenerative or cerebrovascular disease: acute cognitive impairments, psychiatric disorders and functional cognitive disorders may mimic neurodegenerative dementia.(4) Misdiagnoses are known to occur, with dementia diagnoses sometimes being rescinded. The number of dementia diagnoses in healthcare settings therefore reflects the sum of two inputs: the number of progressive dementias, and the number of potentially reversible dementias (see Figure 2). Which of these numbers is being modulated by any theorised risk factor (such as multimorbidity) is not always clear, and may require deliberate research designs to examine.

The cohort described here benefitted from longitudinal follow-up with objective reassessment of cognitive function. We are therefore reasonably confident that dementia diagnoses correspond to sustained, objective impairments. Relatedly, large and population-representative studies with repeated assessment of objective cognitive dysfunction have not supported an association between several physical LTCs and progressive cognitive impairments.(17)

It has been common for large risk factor studies to not objectively assess (and subsequently reassess) dementia, instead deriving this outcome from healthcare records: for example, by examining the first reported onset of dementia,(1) or seeking records of dementia-related healthcare claims.(2) This may raise the risk of including dementia cases with only a transient cognitive impairment alongside those with a progressive dementia.

Such transient or non-progressive dementias will likely have a different aetiology, reflecting causes other than progressive underlying brain pathology. As discussed below, physical and mental factors associated with multimorbidity could be direct causes of transient or non-progressive cognitive symptoms. This might better account for previously reported associations between multimorbidity and dementia in the absence of greater neuropathological change.

Improving the recognition and understanding of such potentially reversible dementias, and any possible links to psychiatric and physical comorbidities, is crucial for future dementia research. Such cases may be present in observational and interventional research studies, particularly those without biomarker or neuropathological confirmation of disease, with important implications for statistical power and interpretation of results.

### Cognitive symptoms and physical comorbidities

We found that physical multimorbidity had a moderating effect of weakening the relationship between AD pathology and clinical dementia; these results are similar in direction and magnitude to reported moderating effects of frailty, a related concept.(18) This effect appeared to be driven by an under-representation of Braak NFT stages V-VI (when there is a high likelihood of cognitive symptoms due to AD) in people with physical multimorbidity, and an over-representation of Braak NFT stages I-II (when AD-related changes have a very low likelihood of causing cognitive symptoms).

This may be explained by the acute or chronic illness directly impairing cognitive performance, mimicking neurodegenerative dementia in the absence of significant pathology. There are several direct consequences of physical multimorbidity which may predispose people to experiencing cognitive symptoms in the absence of dementia-related pathology. In cognitively healthy older adults, physical multimorbidity is associated with greater prevalence of subjective cognitive symptoms(19) – an association mediated by stress, poor sleep, and anxiety. Pain and fatigue, possible consequences of multimorbidity, may also be associated with an FCD-like profile of cognitive symptoms.(20) Polypharmacy is a natural consequence of multimorbidity, with multiple LTCs requiring multiple overlapping treatments. There is a well-recognised association between polypharmacy and cognitive symptoms in later life, particularly when there is an increasing anticholinergic burden.(21)

Subjective, functional, or transient objective cognitive symptoms related to physical comorbidities and polypharmacy could therefore contribute to an increased number of cases with dementia diagnosis in healthcare records.(1) These are likely to not manifest as progressive cognitive impairment sufficient for dementia diagnosis in other settings, and would not be associated with underlying neurodegenerative pathology, potentially explaining divergent findings to date.

We did not find an association between physical multimorbidity and key markers of neuropathological change in this cohort. However, several pathobiological mechanisms could contribute to pathology-related change without being reflected in these neuropathological findings. Synaptic dysfunction/loss, neuroinflammation, mitochondrial dysfunction and cerebral hypoperfusion/hypometabolism are possible contributors to cognitive dysfunction which may not be reflected by neuropathological staging. Any of these could represent important unmeasured mediators between physical multimorbidity and cognitive impairment, requiring further examination.

### Cognitive symptoms and psychiatric comorbidities

Psychiatric comorbidities such as depression may be prodromal features of dementia-related neuropathology, but may also mimic dementia-like cognitive symptoms.(22) This could partially account for previously-reported findings of a link between overall multimorbidity (with previous studies often including mental illnesses) and dementia.

Unlike physical multimorbidity, we found psychiatric multimorbidity to be positively associated with clinical dementia. When co-occurring with Parkinson’s disease, this was explained by underlying Lewy body disease. In the absence of Parkinson’s disease however, this pathological link was not clear. This would be consistent with the dual nature of psychiatric comorbidities as both manifestations and mimics of dementia-related brain changes (see Figure 2). Given the absence of a clear link here between isolated mental health conditions and dementia-related pathology, the observed link between these and dementia seemed mostly unrelated to these being prodromal manifestations of neurodegeneration. This could also suggest a lack of support for hypothesised psychiatric-onset Lewy body disease. However, this warrants further, detailed assessment due to likely heterogeneity. We assessed any reported history of long-term psychiatric conditions: neuropsychiatric and behavioural symptoms of dementia may not necessarily result in such a long-term diagnosis.

While individuals with a cognitive disorder secondary to a psychiatric disorder should not meet consensus criteria for all-cause dementia,(23) misdiagnosis is common. Misdiagnosis of cognitive symptoms in primary psychiatric disorders, or the prodromal manifestation of psychiatric disorders in developing degenerative disease, could therefore partially explain the apparent link between multimorbidity and dementia in this and previous studies. This may be particularly pertinent in younger dementia cohorts (i.e. 60s-70s), when differentiation of dementia from mood disorder is less accurate.

#### Strengths and Limitations

We used data from a clinicopathological study benefitting from comprehensive neuropathological assessment providing gold standard evidence of the presence/absence of dementia-related neuropathology, and drawing from multiple sampling sites to cover regions across England and Wales. This included prospective follow-up of dementia cases and controls. While the overall numbers of participants is smaller than most large epidemiological studies, the number of dementia cases and relative confidence in their diagnoses is a strength.

Drawing an older sample from clinical services and research cohorts, dementia cases in the BDR cohort may have a higher expected prevalence of neurodegenerative changes in contrast to younger population studies. Presence of dementia was assessed through administration of the Clinical Dementia Rating scale within the study by experienced clinical researchers; this is a limitation of this work as the global CDR score is not a diagnostic scale. Final clinicopathological diagnoses were made by an expert clinical panel, however these were not used in this analysis to limit bias from inclusion of postmortem findings in antemortem clinical ratings. While clinical dementia diagnoses may not accurately identify dementia subtype in this cohort, they have previously been shown to be generally accurate as to the presence of dementia-related neuropathological changes overall.(24)

This study was primarily designed to test the association between physical multimorbidity and dementia-related neuropathological change. Detailed assessment of the relationships between multimorbidity, polypharmacy, functional cognitive symptoms and dementia would require carefully designed studies for this specific purpose. Our above explanations are therefore consistent with the data available, but require testing in future studies.

This study did not have data linkage to electronic health records, and multimorbidity was calculated primarily through self-report, supplemented by clinical assessment, with a focus on key age-related diseases. Comorbid conditions could therefore be missed, if not included within the CCI measure, or through not being reported by those with a more severe cognitive impairment (though informants or carers were also interviewed where available).

Individuals who volunteer for prospective research and future brain tissue donation are likely to be healthier than the wider population, which may be a source of bias. While cancer and diabetes were common, other conditions were not, potentially limiting statistical power. Comparable population-representative cohorts have reported higher rates of multimorbidity than found here,(25) though including different indicators of multimorbidity (e.g. hearing impairment). While not fully population-representative, BDR participants came from multiple geographical regions with varying levels of deprivation,(5) which may somewhat attenuate the typical research bias.

Clinically-reported cerebrovascular disease was relatively common in both cognitively impaired and unimpaired groups, contrary to expectations. This may reflect the heterogeneity inherent in cerebrovascular disease as assessed here (which includes strokes, transient ischaemic attacks, and other cerebrovascular events), as well as the poor concordance between clinical and pathological assessment of cerebrovascular disease.

In contrast to previous research, we found no evidence that physical multimorbidity was associated with clinical dementia. However, the majority (56%) of donors with dementia also had one or more comorbid physical LTCs, and therefore met broader criteria for overall multimorbidity since dementia is itself a serious LTC. These comorbidities are likely to impact on quality of life and care in dementia, even if they do not contribute to worse dementia-related neuropathology.

With an average of four years of follow-up before death and an average age at death in the 80s, these data represent associations of later-life multimorbidity, albeit the presence of these morbidities can reach back to earlier life. Previous studies assessed mid-life multimorbidity directly and found this to have a stronger relationship with dementia than late-life multimorbidity.(1) It is therefore possible that these associations shift over time as both multimorbidity and neurodegeneration become more common with increasing age.

This is further complicated by possible survivorship bias: those who develop dementia in later life have not died of another cause earlier, which might induce an apparent negative association where no association exists. Future research including neuropathological assessment may therefore benefit from more comprehensive assessment of comorbid conditions, particularly including their historical presentation, through health record linkage.

#### Conclusions

Previously reported links between physical multimorbidity and dementia are not supported by an association between later-life multimorbidity and greater dementia-related neuropathological change.

## Supplementary Material

Supplementary Table S1

## Figures and Tables

**Table 1:** Demographics of sample, stratified by cognitive status.

	No Dementia,N=328	Dementia,N=439
Age at Death	86 (80, 91)	83 (77, 89)
Age at Baseline	82 (75, 87)	81 (75, 86)
Baseline-Death Delay (Years)	4.8 (2.8, 6.3)	2.9 (1.4, 5.0)
Female Gender	173 (53%)	189 (43%)
Number of Non-Dementia LTCs		
Zero	92 (28%)	194 (44%)
One	135 (41%)	161 (37%)
Two or more	101 (31%)	84 (19%)
Median (IQR); n (%)		

**Table 2:** Rates of each reported long term health condition, stratified by cognitive status.

	No Dementia, N=328	Dementia, N=439
**Primary Physical Conditions**		
Myocardial Infarction	43 (13%)	55 (13%)
Congestive Heart Failure	16 (4.9%)	6 (1.4%)
Peripheral Vascular Disease	19 (5.8%)	9 (2.1%)
Chronic Pulmonary Disease	30 (9.1%)	27 (6.2%)
Rheumatic Disease	18 (5.5%)	8 (1.8%)
Peptic Ulcer Disease	6 (1.8%)	9 (2.1%)
Mild Liver Disease	4 (1.2%)	1 (0.2%)
Diabetes	43 (13%)	55 (13%)
Diabetes with Complications	3 (0.9%)	1 (0.2%)
Hemiplegia	2 (0.6%)	3 (0.7%)
Renal Disease	13 (4.0%)	14 (3.2%)
Cancer	138 (42%)	123 (28%)
Metastatic Cancer	8 (2.4%)	4 (0.9%)
**Secondary Dementia-Related Conditions**		
Parkinson’s Disease	10 (3.1%)	28 (6.5%)
Cerebrovascular Disease	52 (16%)	53 (12%)
Depression	13 (4.0%)	31 (7.2%)
Other Mental Disorder^a^	5 (1.5%)	15 (3.5%)
n (%)		

a Anxiety, Stress, Personality or Psychotic Disorder

## Data Availability

Analytical scripts to replicate these findings are available by request to the corresponding author.

## Relevance Statement

Cognitive impairment in older people has a variety of underlying causes. In addition to neurodegenerative causes such as Alzheimer’s disease, a dementia-like cognitive disorder may appear due to non-degenerative factors.

Multimorbidity has been previously associated with clinical dementia risk, though whether this was due to greater risk of dementia-related neuropathology, or other factors that mimic dementia, was unclear.

We provide evidence that physical multimorbidity is not associated with greater pathological changes at autopsy. Other factors related to multimorbidity and cognitive impairments may be important targets for investigation, such as functional cognitive disorders, primary psychiatric disorders (depression, anxiety, psychosis) and polypharmacy
