# Supplementary material for: Associations between multimorbidity and neuropathology in dementia: a case for considering functional cognitive disorders, psychiatric illness, and dementia mimics
Source: Br J Psychiatry. Author manuscript; Available in PMC 2024 Jun 1. (PMC7615979; doi:10.1192/bjp.2024.25)

Supplementary Table S1. Presence of key neuropathological changes in cohort.

**Thal Phase** (n=499)					
0	***1***	***2***	***3***	***4***	***5***
65 (13%)	69 (14%)	58 (12%)	68 (14%)	102 (20%)	137 (27%)

**Braak Neurofibrillary Tangle Stage** (n=561)						
0	***1***	***2***	***3***	***4***	***5***	***6***
21 (3.7%)	54 (9.6%)	131 (23%)	89 (16%)	41 (7.3%)	71 (13%)	154 (27%)

**CERAD Score** (n=522)			
None	***Sparse***	***Moderate***	***Dense***
155 (30%)	98 (19%)	79 (15%)	190 (36%)

**Braak Lewy body stage** (n=496)						
0	1	2	3	4	5	6
366 (74%)	7 (1.4%)	10 (2.0%)	16 (3.2%)	30 (6.0%)	31 (6.2%)	36 (7.3%)

**Binary Changes Present**			
VCING - Infarcts (n=443)	***VCING - CAA*** (n=442)	***VCING - arteriolosclerosis*** (n=440)	***LATE-NC*** (n=344)
67 (15%)	176 (40%)	109 (25%)	114 (33%)

Supplementary Figure S1. Moderation analysis: presence of physical multimorbidity weakens the association between Alzheimer's pathology and clinical dementia.

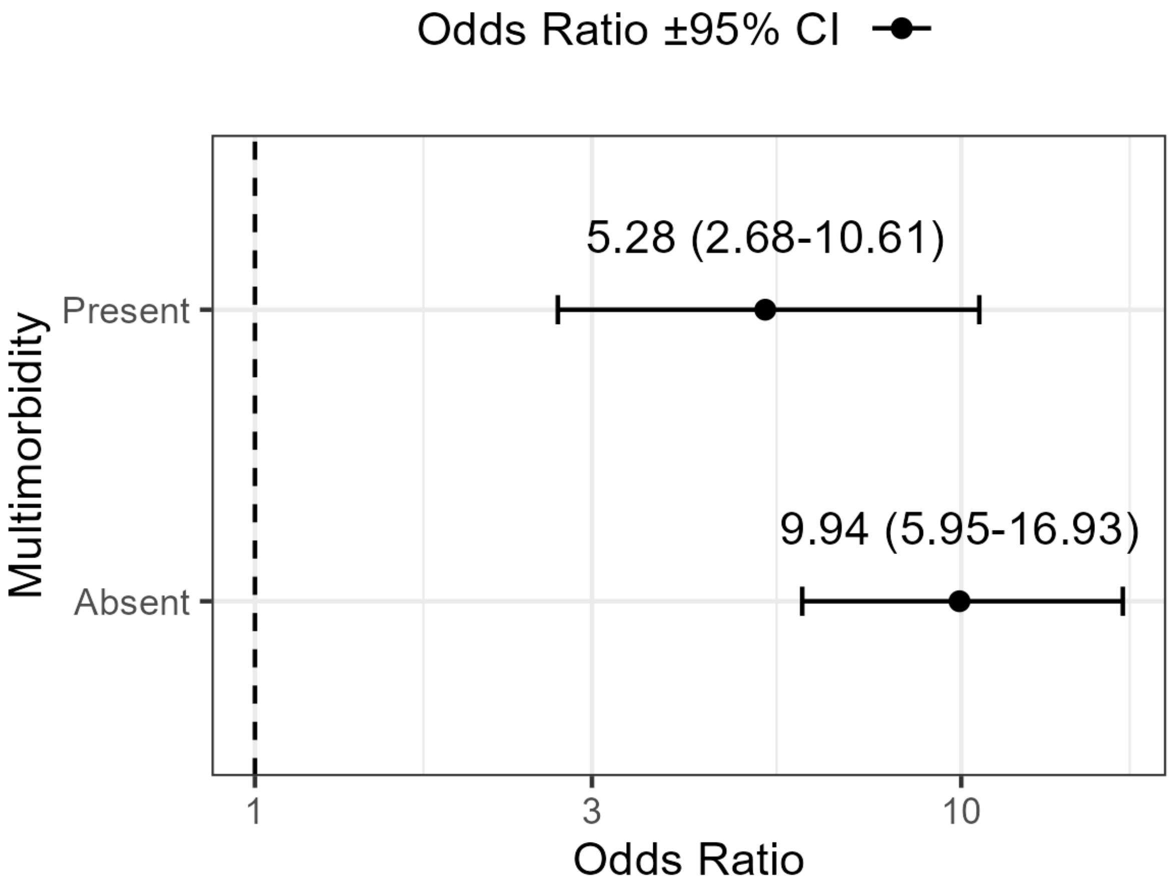

Supplementary Figure S2. Posterior distributions of associations between multimorbidity categories and presence of dementia before death.

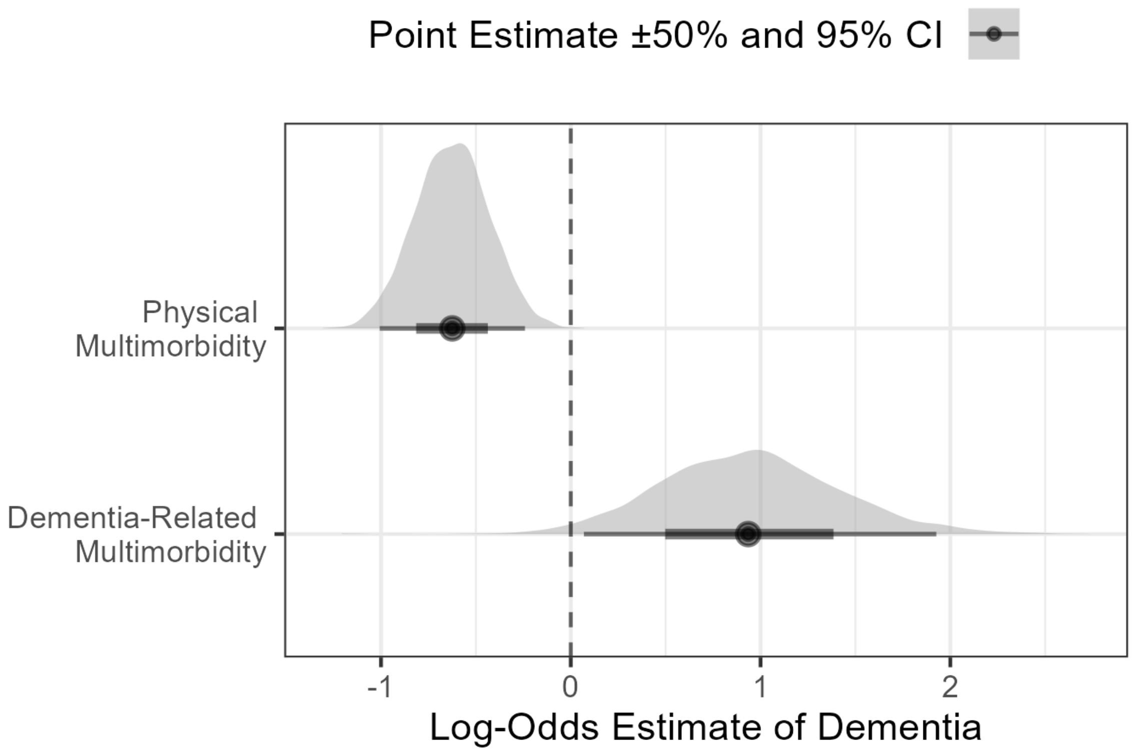

Supplementary Figure S3. Associations of brain comorbidities with dementia and Lewy pathology

Parkinson’s disease, depression, and other mental disorders are associated with dementia (A) and Lewy body disease (B). Psychiatric (multi)morbidity alone is associated with dementia (C) but not Lewy body disease (D).

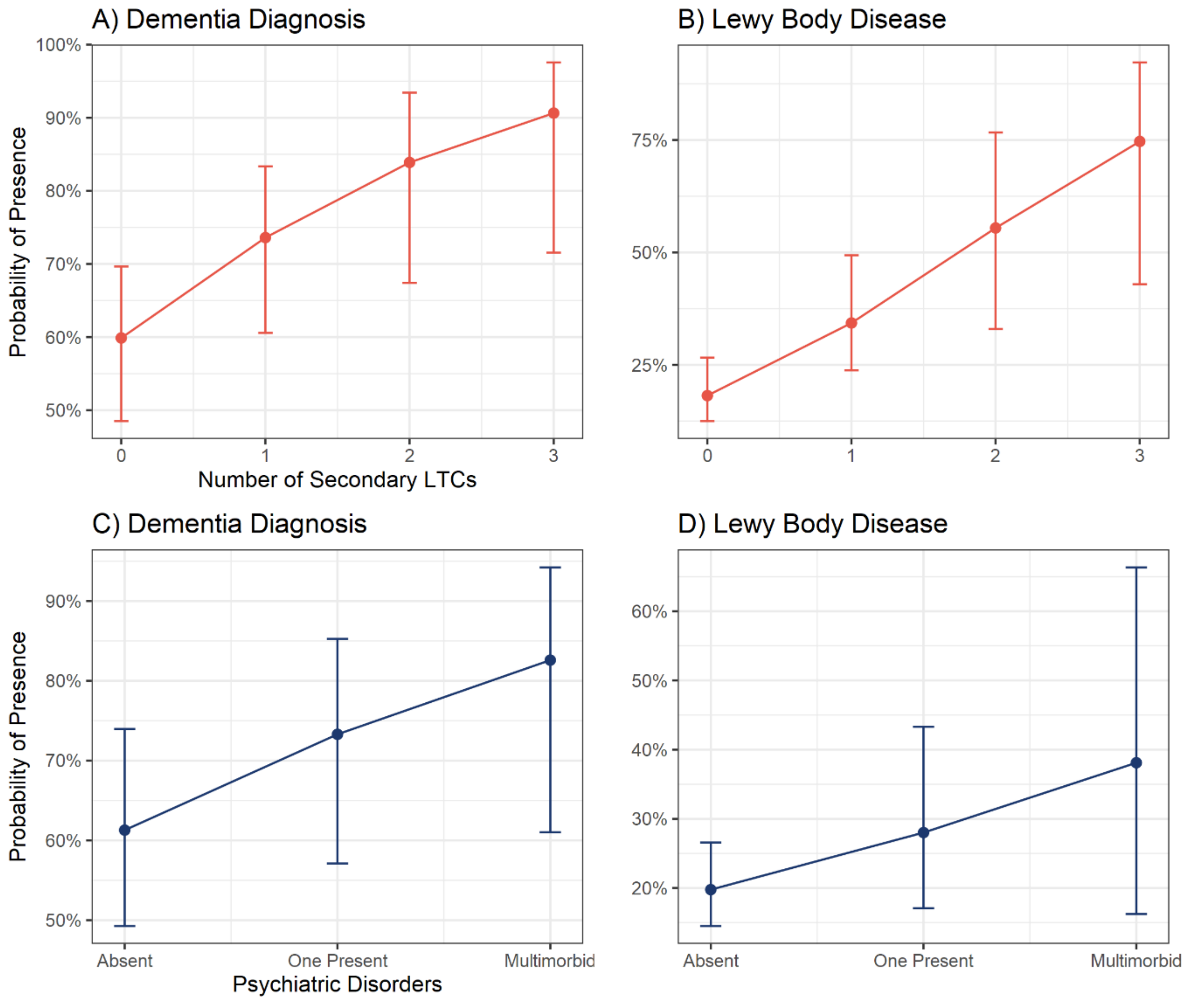

Supplement: Supplementary Table S1 [file EMS193756-supplement-Supplementary_Table_S1.pdf]
